# The effect of the interaction of sleep onset latency and age on ischemic stroke severity via inflammatory chemokines

**DOI:** 10.3389/fneur.2024.1323878

**Published:** 2024-02-16

**Authors:** Yuyu Zhou, Xiaoli Han, Qingshuang Mu, Lifei Xing, Yan Wu, Cunbao Li, Yanlong Liu, Fan Wang

**Affiliations:** ^1^Beijing Hui-Long-Guan Hospital, Peking University, Beijing, China; ^2^Medical Neurobiology Lab, Inner Mongolia Medical University, Huhhot, China; ^3^Clinical Nutrition Department, Friendship Hospital of Urumqi, Urumqi, China; ^4^Xinjiang Key Laboratory of Neurological Disorder Research, The Second Affiliated Hospital of Xinjiang Medical University, Urumqi, China; ^5^Department of Neurology, Sinopharm North Hospital, Baotou, China; ^6^School of Mental Health, Wenzhou Medical University, Wenzhou, China

**Keywords:** sleep onset latency, age, ischemic stroke, inflammation, interaction

## Abstract

**Objective:**

Prolonged sleep onset latency (PSOL) and age have been linked to ischemic stroke (IS) severity and the production of chemokines and inflammation, both of which contribute to IS development. This study aimed to explore the relationship between chemokines, inflammation, and the interplay between sleep onset latency (SOL) and age in influencing stroke severity.

**Methods:**

A cohort of 281 participants with mild to moderate IS was enrolled. Stroke severity was assessed using the National Institutes of Health Stroke Scale (NIHSS), and SOL was recorded. Serum levels of macrophage inflammatory protein-1alpha (MIP-1α), macrophage inflammatory protein-1beta (MIP-1β), monocyte chemoattractant protein-1 (MCP-1), interleukin-6 (IL-6), and tumor necrosis factor-alpha (TNF-α) were measured.

**Results:**

NIHSS scores of middle-aged participants with PSOL were significantly higher than those with normal sleep onset latency (NSOL) (*p* = 0.046). This difference was also observed when compared to both the elderly with NSOL (*p* = 0.022), and PSOL (*p* < 0.001). Among middle-aged adults with PSOL, MIP-1β exhibited a protective effect on NIHSS scores (β = −0.01, *t* = −2.11, *p* = 0.039, *R*^2^ = 0.13). MIP-1α demonstrated a protective effect on NIHSS scores in the elderly with NSOL (β = −0.03, *t* = −2.27, *p* = 0.027, *R*^2^ = 0.12).

**Conclusion:**

This study reveals a hitherto undocumented association between PSOL and IS severity, along with the potential protective effects of MIP-1β in mitigating stroke severity, especially among middle-aged patients.

## 1 Introduction

Stroke is considered the second leading cause of death worldwide and remains a significant cause of disability in both developed and developing countries (1). Ischemic stroke accounts for almost 70% of all stroke cases (1). There is a rich literature available substantiating that prolonged sleep onset latency (PSOL) and age can determine stroke severity (2–4). However, the precise mechanisms by which PSOL and age impact the development of ischemic stroke (IS) remain incompletely understood.

Sleep onset latency (SOL) is the amount of time it takes a person to fall asleep in bed, and represents an important marker for assessing sleep quality (5). PSOL is one of the main manifestations of sleep structural changes in ischemic stroke (6). Studies have demonstrated a positive correlation between PSOL and the prevalence of stroke; short SOL was associated with a 36% reduction in the risk of stroke, while PSOL was also related to the severity of IS symptoms (7), suggesting that shorter SOL may protect against stroke (2). In addition, it has been shown that sleep onset latency is prolonged with aging (8), and aging is a significant factor affecting stroke (3). Besides, stroke tends to occur predominantly in the elderly, and its incidence and severity are closely related to age (3, 9, 10). Consistently, a study revealed that the severity of strokes tended to increase with increasing age (10). In contrast, a growing body of evidence suggests the “younger stroke” phenomenon is gaining prominence as a pressing public health issue, marked by a rising occurrence of strokes among individuals considered “younger” (those under 50 years of age) (11, 12). Consequently, SOL and age have been established as risk factors for ischemic stroke. Nonetheless, the underlying pathophysiological mechanisms governing their interplay in influencing the severity of IS remain uncertain.

Prolonged sleep onset latency can lead to a series of sleep-related issues that exacerbate stroke severity by triggering a systemic inflammatory response (13–16). In addition, prior investigations have shown that PSOL is exacerbated with age (17). However, recent research has indicated prolonged sleep onset latency even among middle-aged individuals, indicating a close relationship between SOL and age (18). Interestingly, age also impacts stroke severity through its influence on inflammation (19). Chemokines, as small molecular proteins, play a crucial role in the immune and inflammatory responses after stroke, which are involved in the processing of neovascularization, neurogenesis, and neural network reconstruction (20). Chemokines are cytokines attracting selective leukocyte subsets and subgrouping into the four major subfamilies, CC, CXC, C, and CX3C. macrophage inflammatory protein-1 alpha (MIP-1α), macrophage inflammatory protein 1beta (MIP-1β), and monocyte chemoattractant protein-1 (MCP-1) are the three best-known and most extensively studied CC chemokines in primary and secondary inflammatory responses in humans (21, 22). An increasing body of literature suggests that chemokines and cytokines, such as high levels of MIP-1α, MIP-1β, MCP-1, interleukin-6 (IL-6) and tumor necrosis factor-alpha (TNF-α) are associated with poor subjective sleep quality characterized by PSOL (23–27). In this respect, animal experiments have demonstrated that older mice exhibit notably higher MIP-1α and MIP-1β levels than their younger counterparts (28), which indicate that the cytokines and chemokines are also closely related to age. The overexpression of chemokines MIP-1α and MCP-1 can promote the recruitment of inflammatory cytokines IL-6 and TNF-α (29), and the recruitment of pro-inflammatory factors accelerates the development of atherosclerotic plaques, which further aggravates blood–brain barrier injury (30) and leads to brain injury. The chemokine-induced inflammatory response is pivotal in exacerbating stroke outcomes (30, 31). These cumulative factors collectively contribute to the heightened severity of ischemic stroke (32, 33). In essence, the combined influence of sleep onset latency and age on these cytokines may provide insights into explaining the underlying pathophysiology of ischemic stroke.

As described above, most studies have shown that SOL and age are independently correlated with stroke severity (2, 3, 7), and these cytokines played roles in the severity of IS. However, the association between chemokines, inflammation, and the interaction of SOL and age with stroke severity remains elusive, yet it holds crucial significance for preventing ischemic stroke. Therefore, this study aimed to examine how the interplay between sleep onset latency and age impacts chemokine levels and inflammation, with a subsequent exploration of their combined role in determining the severity of strokes.

## 2 Materials and methods

### 2.1 Participants

A total of 281 participants with mild and moderate ischemic stroke admitted to Sinopharm North Hospital from June 2020 to December 2021 were recruited.

Sociodemographic data, such as age, years of education, occupation, and current body mass index (BMI), were collected. Clinical data, such as a history of substance abuse and dependence, were obtained according to medical records and self-reports and confirmed by the next of kin and family members. Data on SOL in the 1–3 months before stroke were collected by self-assessment and report.

The following criteria were used for participant inclusion: individuals aged 45–80 diagnosed with mild and moderate ischemic stroke based on clinical symptoms, physical examination, and imaging findings. Participants with a history of working night shifts, diagnosed with severe stenosis of the internal carotid artery, external carotid artery, subclavian artery, and vertebral artery as evident by cranial MRA and vascular color ultrasound, individuals diagnosed with tumors, those experiencing significant and persistent sleep problems along with diagnosed sleep disorders, or those taking medications and healthcare products known to affect sleep patterns were excluded. In addition, participants with severe and very severe ischemic stroke were excluded due to the high prevalence of altered consciousness, such as coma, which would hinder the accurate assessment of sleep patterns. The exclusion criteria also included a history of any substance abuse or dependence, as well as any neurological and psychiatric disorders diagnosed by the Statistical Manual of Mental Disorders-V (DSM-V).

The present study was approved by the Institutional Review Board of the Sinopharm North Hospital (Approval number: GYBFYY-LL-2020006) and was performed in accordance with the Declaration of Helsinki, and written informed consent was obtained. No financial compensation was provided to the subjects in this study.

### 2.2 Assessments and laboratory tests

The National Institutes of Health Stroke Scale (NIHSS) contains 15 items, a reliable, valid, and responsive tool for measuring stroke severity (34). The NIHSS includes the following domains: level of consciousness, eye movements, integrity of visual fields, facial movements, arm and leg muscle strength, sensation, coordination, language, speech, and neglect. Each impairment is scored on an ordinal scale ranging from 0 to 2, 0 to 3, or 0 to 4. The cumulative scores yield a total ranging from 0 to 42, with higher scores indicating more severe strokes (35). Stroke severity was categorized as follows: mild (NIHSS score 0–5), moderate (NIHSS score 6–14), severe (NIHSS score 15–24), and very severe (NIHSS score 25) (36, 37).

Recognizing that sleep onset latencies exceeding 30 min are associated with sleep difficulties in middle-aged and older adults (38), the present study categorized participants based on this established criterion (38). Participants with a sleep onset latency of more than 30 min were grouped as the PSOL group (*n* = 153), and those who had an SOL of 30 min or less constituted the normal sleep onset latency (NOSL) group (*n* = 127).

High-density lipoprotein (HDL), low-density lipoprotein (LDL), total cholesterol (TC), and triglyceride (TG) levels were obtained from routine tests to assess the participants’ physical condition in relation to ischemic stroke. SOL data and NIHSS scores were collected after peripheral metabolic markers were measured on the first day of admission. Participants were admitted to the hospital either on the day of the onset of physical symptoms or the next day.

Peripheral blood samples were obtained upon admission. The serum was separated and immediately frozen at −80°C. Analyses were performed to measure the serum levels of MIP1α, MIP1β, MCP1, IL-6, and TNFα using ELISA kits (Shanghai Xinle Biotechnology Co., LTD, Shanghai, China). Laboratory technicians conducting the analyses were blinded to clinical data.

### 2.3 Statistical analysis

Data were presented as mean ± standard deviation (SD) for continuous variables and as frequencies and percentages for categorical variables. The comparison of categorical variables was performed by the chi-squared test. The normality of all variables was assessed using the Shapiro–Wilk test. Levene’s test verified the homoscedasticity of residual variances, confirming the equal distribution of residuals (all *p* > 0.05). As a result, an analysis of covariance (ANCOVA) was employed to compare differences in inflammatory markers between groups (see Table 1). Partial correlation analysis was used to examine the correlation between inflammatory markers and NIHSS scores.

**Table 1:** The differences in clinical characteristics between groups.

Variables	PSOL (>30 min)		NSOL (≤30 min)		*F*/χ^2^	*p*
	Middle-aged (*n* = 62)	Elderly (*n* = 91)	Middle-aged (*n* = 59)	Elderly (*n* = 68)		
Age (years)	57.29 ± 5.03	72.29 ± 4.65	57.24 ± 4.69	71.93 ± 4.06	283.60	<0.001***
Gender					24.64	<0.001***
Male	37 (59.7%)	39 (42.9%)	49 (83.1%)	44 (63.8%)		
Female	25 (40.3%)	52 (57.1%)	10 (16.9%)	25 (36.2%)		
BMI (Kg/m^2^)	25.38 ± 2.76	24.88 ± 3.39	24.90 ± 3.05	25.00 ± 2.75	0.37	0.766
Education (years)	8.97 ± 2.90	6.35 ± 3.26	9.02 ± 3.16	7.06 ± 3.13	13.41	<0.001***
Active drinker					23.54	<0.001***
Yes	22 (35.5%)	15 (16.5%)	30 (50.8%)	15 (21.7%)		
No	40 (64.5%)	76 (83.5%)	29 (49.2%)	54 (78.3%)		
Active smoker					28.02	<0.001***
Yes	33 (53.2%)	28 (30.8%)	44 (74.6%)	33 (47.8%)		
No	29 (46.8%)	63 (69.2%)	15 (25.4%)	36 (52.2%)		
Hypertension					6.28	0.100
Yes	43 (69.4%)	65 (71.4%)	40 (67.8%)	36 (52.9%)		
No	19 (30.6%)	26 (28.6%)	19 (32.2%)	32 (47.1%)		
Diabetes					6.48	0.090
Yes	15 (24.2%)	33 (36.3%)	11 (18.6%)	17 (24.6%)		
No	47 (75.8%)	58 (63.7%)	48 (81.4%)	52 (75.4%)		
Hyperlipidemia					2.46	0.482
Yes	21 (33.9%)	22 (24.2%)	14 (23.7%)	21 (30.9%)		
No	41 (66.1%)	69 (75.8%)	45 (76.3%)	47 (69.1%)		
HDL (mmol/L)	1.14 ± 0.23	1.17 ± 0.27	1.16 ± 0.28	1.14 ± 0.26	0.18	0.913
LDL (mmol/L)	3.14 ± 0.74	3.27 ± 2.35	2.96 ± 0.94	2.96 ± 1.00	0.73	0.537
TC (mmol/L)	4.82 ± 1.04	4.61 ± 1.20	4.49 ± 1.22	4.44 ± 1.19	1.26	0.290
TG (mmol/L)	2.37 ± 1.59	1.60 ± 0.75	1.83 ± 0.86	1.88 ± 1.33	5.20	0.002**
MIP-1α (ng/L)	53.86 ± 28.11	49.90 ± 22.26	47.29 ± 19.83	48.80 ± 21.34	0.78	0.511
MIP-1β (ng/L)	158.43 ± 72.09	145.53 ± 66.19	142.80 ± 61.16	133.03 ± 53.19	1.61	0.188
MCP-1 (ng/L)	157.44 ± 69.21	141.55 ± 65.80	138.84 ± 59.92	141.21 ± 75.02	0.89	0.445
IL-6 (ng/L)	111.43 ± 47.92	107.55 ± 50.02	92.60 ± 32.78	99.37 ± 38.38	2.19	0.089
TNFα (ng/L)	97.71 ± 46.36	91.97 ± 41.65	87.96 ± 47.31	96.10 ± 42.04	0.55	0.645

PSOL, prolonged sleep onset latency; NSOL, normal sleep onset latency; BMI, body mass index; HDL, High-density lipoprotein; LDL, Low-density lipoprotein; TC, total cholesterol; TG, triacylglycerol; MIP-1α, macrophage inflammatory protein-1alpha; MIP-1β, macrophage inflammatory protein-1beta; MCP-1, monocyte chemoattractant protein-1; IL-6, interleukin-6; TNFα, tumor necrosis factor-alpha.

Data were presented as mean ± standard deviation (SD) for continuous variables and as frequencies and percentages for categorical variables. *p* value for analysis of covariance (ANCOVA) or chi-square test, *p < 0.05, **p < 0.01, ***p < 0.001.

In addition, general linear models (GLMs) were applied to test the significance of the interaction between SOL and age and their effect on NIHSS scores. Current BMI was included as a covariate in all models. Model comparisons and testing were carried out using an F-statistic.

All statistical analyses were performed using IBM SPSS Statistics for Windows, Version 22.0 (IBM Corp., Armonk, NY, United States). Figures were generated using GraphPad Prism version 8 (GraphPad Software Inc.). All tests were two-sided, and the significance threshold was set at *p* < 0.05.

## 3 Results

### 3.1 Demographic and clinical characteristics

An ANCOVA was conducted with BMI as the covariate to identify disparities in sociodemographic, clinical variables, and inflammatory markers across various groups (Table 1). In contrast to participants with NSOL, those with PSOL exhibited a higher proportion of females (50.7% vs. 27.1%, *p* < 0.001). Participants in the PSOL group reported lower rates of smoking than those in the NSOL group (39.5% vs. 60.5%, *p* < 0.001), while no difference was observed in other sociodemographic and clinical characteristics between both groups.

### 3.2 Analysis of differences between groups

The participants were divided into age groups [Middle-aged (aged 45–65) and Elderly (aged 65+)] and presence of PSOL/NSOL which resulted in four distinct groups:Middle-aged with PSOL (*n* = 62), Middle-aged with NSOL (*n* = 59), Elderly with PSOL (*n* = 91), and Elderly with NSOL (*n* = 68).

The homogeneity of variance for the NIHSS scores variable, determined through Levene’s test, yielded a *p*-value greater than 0.05. Thus, ANCOVA was employed to compare differences in NIHSS scores between the groups. Taking current BMI as the covariate, the impacts of SOL and age on NIHSS scores were found to be significant (*F* = 6.51, *p* = 0.011). In this regard, the NIHSS scores in the Middle-aged with PSOL group were notably higher than those in both the Elderly with NSOL group and the Elderly with PSOL group (*p* = 0.015 and *p* < 0.001, respectively).

### 3.3 General linear models analysis

**Figure 1:**
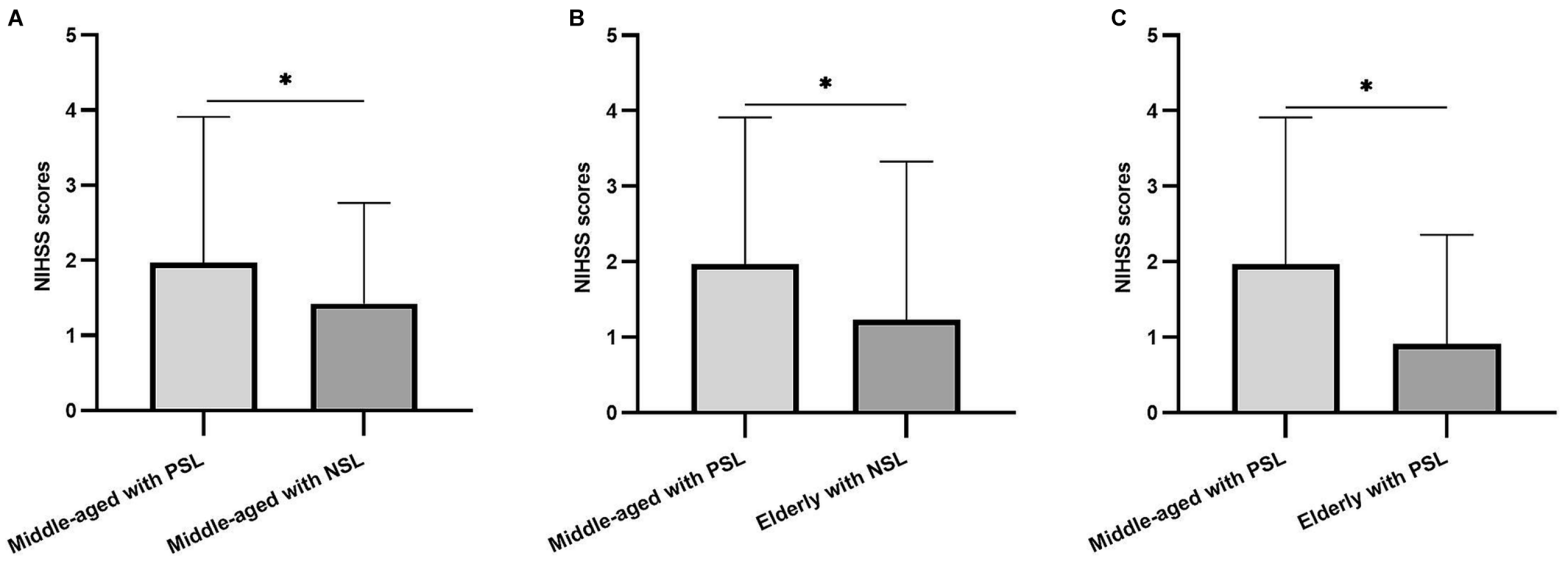
The difference in National Institutes of Health Stroke Scale (NIHSS) scores between groups. **(A)** The differences among the NIHSS scores of the participants in Middle-aged with prolonged sleep onset latency (PSOL) and Middle-aged with normal sleep onset latency (NSOL); **(B)** The differences among the NIHSS scores of the participants in Middle-aged with PSOL and Elderly with NSOL; **(C)** The differences among the NIHSS scores of the participants in Middle-aged with PSOL and Elderly with PSOL. *p < 0.05.

To explore potential interactions between SOL and age in relation to stroke severity, GLM analyses of NIHSS scores were performed while controlling for current BMI. GLM analysis revealed strong interactions for NIHSS scores between SOL and age within the dataset. Notably, the NIHSS scores of participants in the Middle-aged with PSOL group were significantly higher compared to the Middle-aged with NSOL group (*p* = 0.046). Furthermore, the NIHSS scores of participants in the Middle-aged with PSOL group were significantly elevated compared to those in both the Elderly with NSOL group (*p* = 0.022) and the Elderly with PSOL group (*p* < 0.001; Table 2, Figure 1).

**Table 2:** The interaction of SOL and age on NIHSS scores.

Variables	PSOL (>30 min)		NSOL (≤30 min)		MD	*p*
	Middle-aged (*n* = 62)	Elderly (*n* = 91)	Middle-aged (*n* = 59)	Elderly (*n* = 68)		
	Mean ± SD	Mean ± SD	Mean ± SD	Mean ± SD		
NIHSS scores	1.95 ± 0.21	–	1.31 ± 0.23	–	0.64	0.046*
	1.95 ± 0.21	–	–	1.26 ± 0.20	0.69	0.022*
	1.95 ± 0.21	0.90 ± 0.17	–	–	1.05	<0.001***

PSOL, prolonged sleep onset latency; NSOL, normal sleep onset latency; NIHSS, National Institutes of Health Stroke Scale; GLM, general linear models; MD, Mean differences; SD, standard deviation.

GLM was used to calculate the differences in levels between four groups with BMI as the covariate. The simple effect was calculated using GLM. Data were reported as mean ± SD, *p < 0.05, **p < 0.01, ***p < 0.001.

### 3.4 Correlations analysis

**Figure 2:**
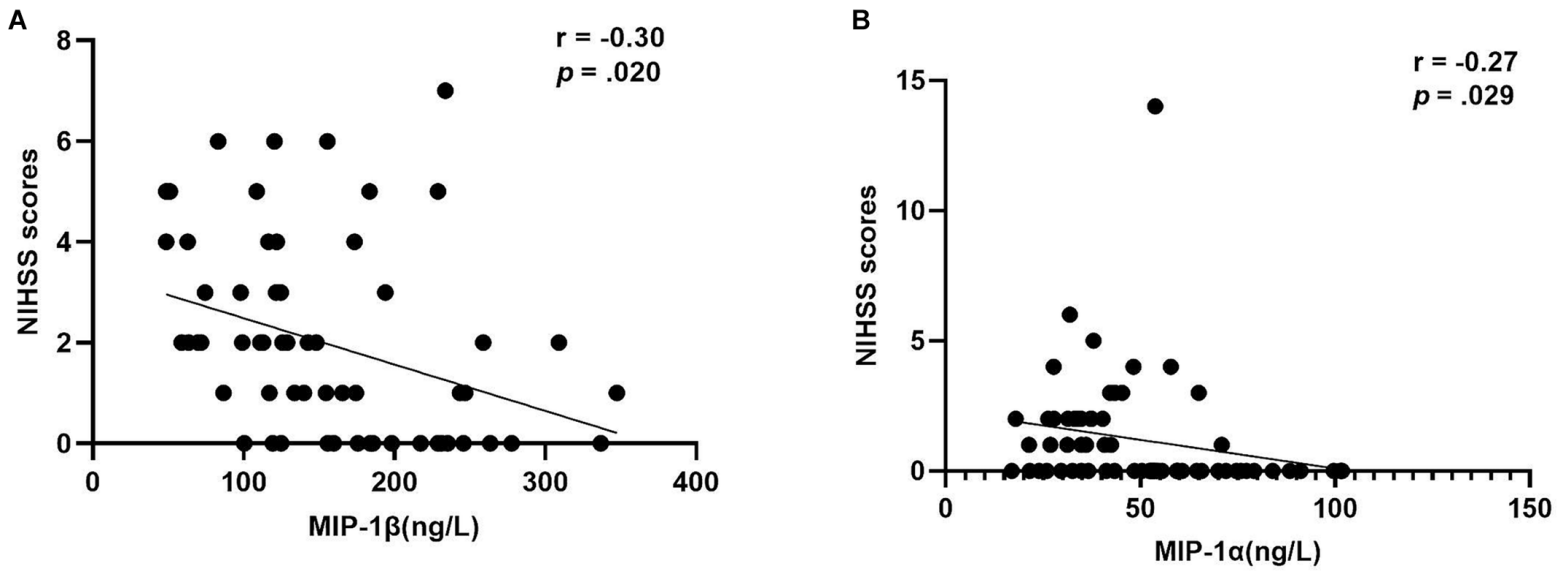
The correlation of MIP-1β levels and MIP-1α levels with NIHSS scores. **(A)** The negative correlation of macrophage inflammatory protein 1beta (MIP-1β) levels with the NIHSS scores (*r* = −0.30, *p* = 0.020) in group of middle-aged with PSOL; **(B)** The negative correlation of macrophage inflammatory protein-1 alpha (MIP-1α) levels with the NIHSS Scores (*r* = −0.27, *p* = 0.029) in the group of Elderly with NSOL.

After adjusting for current BMI, a partial correlation analysis was conducted to assess the relationship between NIHSS scores and inflammatory markers within each group. Notably, a negative correlation was observed between MIP-1β levels and NIHSS scores in the Middle-aged with PSOL group (*r* = −0.30, *p* = 0.020). Similarly, a negative correlation was found between MIP-1α levels and NIHSS scores in the Elderly with NSOL group (*r* = −0.27, *p* = 0.029; Table 3, Figure 2).

**Table 3:** Correlation between NIHSS scores and inflammatory cytokines in different groups.

Groups correlation		MIP-1α (ng/L)	MIP-1β (ng/L)	MCP-1 (ng/L)	IL-6 (ng/L)	TNFα (ng/L)
Middle-aged with NSOL	*r*	−0.15	−0.20	−0.05	0.13	0.06
	*p*	0.290	0.143	0.735	0.343	0.691
Middle-aged with PSOL	*r*	−0.22	−0.30	−0.09	−0.06	0.02
	*p*	0.096	0.020*	0.494	0.678	0.860
Elderly with NSOL	*r*	−0.27	−0.18	−0.22	0.08	0.07
	*p*	0.029*	0.152	0.087	0.525	0.609
Elderly with PSOL	*r*	−0.01	−0.14	0.05	0.07	0.18
	*p*	0.895	0.203	0.667	0.547	0.099

PSOL, prolonged sleep onset latency; NSOL, normal sleep onset latency; MIP-1α, macrophage inflammatory protein-1alpha; MIP-1β, macrophage inflammatory protein-1beta; MCP-1, monocyte chemoattractant protein-1; IL-6, interleukin-6; TNFα, tumor necrosis factor-alpha.

Correlations between MIP-1α, MIP-1β, MCP-1, IL-6, TNFα levels and NIHSS scores were calculated using Partial correlation. *p < 0.05, **p < 0.01.

### 3.5 Hierarchical stepwise linear regression analysis

A hierarchical stepwise linear regression analysis revealed noteworthy findings, with BMI as the initial covariate and NIHSS score as the dependent variable. MIP-1β levels emerged as a protective factor for NIHSS scores in Middle-aged adults with PSOL (β = −0.01, 95%CI [−0.01 ~ 0.00], *t* = −2.11, *p* = 0.039, *R*^2^ = 0.13). Additionally, MIP-1α levels were identified as a protective factor for NIHSS scores in the Elderly with NSOL group (β = −0.03, 95%CI [−0.05 ~ 0.00], *t* = −2.27, *p* = 0.027, *R*^2^ = 0.12).

## 4 Discussion

This pioneering study aims to shed light on the hitherto underexplored relationship between sleep onset latency, age, and stroke severity by investigating the pathophysiological mechanisms potentially driving this association. Importantly, we substantiated the association between PSOL and the severity in middle-aged IS participants, with higher NIHSS scores associated with PSOL and lower levels of MIP-1β.

Our findings suggest that middle-aged stroke participants with PSOL are at greater risk of experiencing a severe stroke, and MIP-1β plays a protective role against IS. Over the years, studies have emphasized that high-risk factors for stroke occurrence (39) and increased stroke severity (5, 10, 40) include sleep difficulties and advanced age. Notably, while stroke has conventionally been linked to older age, recent years have witnessed a substantial decline in the average age of stroke onset, coupled with a rise in stroke incidence and hospitalization rates among middle-aged individuals. This phenomenon of “younger-age stroke” has emerged as a significant public health challenge (12, 41–43), consistent with the results of this study. Indeed, middle-aged people with PSOL face an elevated risk of more severe strokes, possibly attributable to several factors. Firstly, compared to older individuals, middle-aged individuals necessitate efficient and higher sleep quality to sustain bodily functions and metabolism (44–46). Hence, when middle-aged stroke patients with PSOL experience a range of sleep-related issues such as diminished sleep quality, insomnia, and inadequate sleep (15), their sleep requirements are unmet, significantly impeding the recovery from cerebral ischemia-induced reversible or irreversible synaptic and membrane failures, which, influences neuroplasticity and post-stroke recovery (47). Secondly, older individuals usually have more flexible morning routines due to retirement, alleviating the impact of PSOL-related sleep shortage (18). Conversely, middle-aged individuals contend with heightened work pressures, constrained wake-up times and are more prone to insufficient sleep and subpar sleep quality (18). Moreover, middle-aged individuals tend to engage in more social activities, potentially adopting unhealthy lifestyles like high-calorie diets, smoking, and alcohol consumption (48). Besides, the compounded effects of sleep deprivation, stress, and unhealthy habits are widely acknowledged to exacerbate stroke severity (49–51).

In addition, this study found that MIP-1β was negatively associated with NIHSS scores in the middle-aged group with PSOL, indicating that elevated levels of MIP-1β could protect against severe strokes in this cohort. Previous research has indicated the potential involvement of MIP-1β in monocyte recruitment within atherosclerotic plaques, where heightened serum MIP-1β levels have been associated with the progression of IS (52). Despite the established association between PSOL and stroke severity, our study suggests that elevated serum MIP-1β levels could potentially mitigate the severity of stroke events in middle-aged patients with PSOL. Several underlying mechanisms could account for this phenomenon. First, the mRNA and protein expression of the chemokine MIP-1β has been reported to be inhibited by prostaglandin E2 (PGE2) (53). PGE2, a pivotal endogenous anti-inflammatory mediator linked to sleep regulation, demonstrates wakefulness-promoting properties (53). Notably, its concentration is markedly higher during wakefulness compared to slow-wave sleep (54). In this context, participants grappling with PSOL are prone to extended periods of wakefulness (15), leading to heightened PGE2 levels and diminished MIP-1β levels. Intriguingly, PGE2’s impact extends further, potentially playing a dual role. PGE2 has been identified as a disruptor of Na(+)-Ca(2+) exchange and Ca(2+) homeostasis through the EP1 receptor, thereby contributing to excessive Ca(2+) accumulation. This effect also extends to the induction of neuronal cell death and the augmentation of ischemic-induced neurodegeneration (55), ultimately amplifying stroke severity. Indeed, it is highly conceivable that the deleterious influence of MIP-1β on stroke severity in middle-aged individuals with PSOL might be attenuated by PGE2, potentially even manifesting as a protective function. However, this study did not observe a correlation between serum MIP-1β levels and NIHSS scores in older adults with PSOL, attributed to the confounding impact of age. In this regard, one animal study unveiled heightened expression of MIP-1β levels in older mice (28), while another investigation highlighted an accelerated decline in serum MIP-1β levels with aging (56). Consequently, aging appears to disrupt the relationship between MIP-1β and NIHSS scores in older individuals with PSOL. In essence, the protective role of MIP-1β against stroke severity seems to be confined to middle-aged patients with PSOL.

Besides, the NSOL group displayed a potential protective effect against stroke in the elderly, as evidenced by the negative association between MIP-1α and NIHSS scores. Previous studies have shown that, compared to middle-aged mice, chemokine MIP-1α levels are highly expressed in older mice (28). Interestingly, it has been found that MIP-1α levels were significantly reduced in the brain tissue of older patients with IS (57), and MIP-1α tended to decline with age in this patient population (56). At the same time, the NIHSS score at admission increased significantly with age (58). Therefore, the negative correlation between serum MIP-1α level and NIHSS score in senile stroke patients with NSOL may be due to aging. However, our study did not observe the correlation between serum MIP-1α and NIHSS score in elderly patients with PSOL, attributed to the fact that individuals suffering from PSOL exhibited suboptimal sleep quality, consequently experiencing extended periods of wakefulness, which led to an increase in PGE2. The increased PGE2 led to a decline in the chemokine MIP-1α level (59), thus disrupting the age-related negative correlation typically observed between serum MIP-1α levels and NIHSS scores.

Besides, we found no association between MCP-1, IL-6, and TNF-α and NIHSS scores across the four groups. However, prior research on individuals with severe IS demonstrated a correlation between elevated levels of these factors and the severity of IS (60–62). Discrepancies in outcomes might stem from the inclusion of subjects with mild to moderate IS in this current study. Moreover, a prior investigation examining stroke severity 7 days after admission demonstrated a positive association between elevated MCP-1 levels and heightened stroke severity at the same time point (61), Conversely, the present study did not reveal a connection between MCP-1 and stroke severity. This discrepancy may be attributable to the timing of MCP-1 measurement; in this study, samples were collected on day one after admission, whereas the previous study assessed MCP-1 levels 7 days post-stroke. The temporal dynamics of ischemic brain cell damage likely influence the correlation between MCP-1 and stroke severity. In cases where initial ischemic attack results in more severe injury, cytokine and chemokine production may be suppressed. This offers a potential explanation for the absence of a correlation between MCP-1 and NIHSS scores in the current study (61).

Several limitations need consideration within this study. Firstly, the participant pool only comprised Chinese individuals residing in the northern inland region, regardless of whether they were experiencing their first episode or recurrence. Future research should prioritize geographically diverse recruitment and larger sample sizes to improve generalizability of results. Additionally, it is essential to differentiate between first-episode patients and those with recurrent episodes and to conduct stratification analyses based on the number of episodes to bolster result accuracy. Secondly, since sleep patterns influence IS over an extended duration, this study only retrospectively gathered SOL data from 1 to 3 months preceding the IS onset. While data from this brief interval might not comprehensively capture the impact, collecting recent-stage sleep data retrospectively was more feasible, and patient cooperation was facilitated. Lastly, most IS participants in this study exhibited mild to moderate stroke. Consequently, it is important to acknowledge that the generalizability of our study findings may be limited to cases of milder stroke severity. Nevertheless, the presented results provide valuable guidance for the development of targeted preventive interventions for individuals at risk of such strokes.

## 5 Conclusion

The present study provides strong evidence of the association between PSOL and the severity of IS and the potential protective effects of MIP-1β in reducing stroke severity, especially in middle-aged patients, suggesting that falling asleep quickly might contribute to low ischemic stroke severity. In the future, the role of other subfamilies of chemokines in the interaction of sleep onset latency and age on IS severity should be further explored to improve and supplement this study.

## Data availability statement

The original contributions presented in the study are included in the article/supplementary material, further inquiries can be directed to the corresponding authors.

## Ethics statement

The studies involving humans were approved by the Institutional Review Board of the Sinopharm North Hospital (Approval number: GYBFYY-LL-2020006). The studies were conducted in accordance with the local legislation and institutional requirements. The participants provided their written informed consent to participate in this study.

## Author contributions

FW: Conceptualization, Funding acquisition, Writing – review & editing. YZ: Conceptualization, Data curation, Writing – original draft. XH: Writing – original draft. QM: Writing – original draft. LX: Methodology, Writing – review & editing. YW: Data curation, Funding acquisition, Writing – review & editing. CL: Conceptualization, Writing – review & editing, Methodology. YL: Conceptualization, Writing – review & editing, Methodology.

## Figures and Tables

**Table:** 

BMI	Body mass index
GLM	General linear models
HDL	High-density lipoprotein
IL-6	Interleukin-6
IS	Ischemic stroke
LDL	Low-density lipoprotein
MIP-1α	Macrophage inflammatory protein-1alpha
MIP-1β	Macrophage inflammatory protein-1beta
MCP-1	Monocyte chemoattractant protein-1
MD	Mean differences
NIHSS	National Institutes of Health Stroke Scale
NSOL	Normal sleep onset latency
PSOL	Prolonged sleep onset latency
SOL	Sleep onset latency
SD	Standard deviation
TNFα	Tumor necrosis factor-alpha
TC	Total cholesterol
TG	Triacylglycerol

## References

[1] Song Y Zhang X Li C Xu S Zhou B Wu X. Is bilirubin associated with the severity of ischemic stroke? A dose response Meta-analysis. J Clin Med. (2022) 11:3262. doi: 10.3390/jcm11123262, PMID: 35743332 PMC9224549

[2] Kadier K Qin L Ainiwaer A Rehemuding R Dilixiati D Du YY. Association of Sleep-Related Disorders with cardiovascular disease among adults in the United States: a cross-sectional study based on National Health and nutrition examination survey 2005-2008. Front Cardiovasc Med. (2022) 9:954238. doi: 10.3389/fcvm.2022.954238, PMID: 35990939 PMC9386143

[3] Guo Y Wang H Tian Y Wang Y Lip GY. Multiple risk factors and Ischaemic stroke in the elderly Asian population with and without atrial fibrillation. An analysis of 425,600 Chinese individuals without prior stroke. Thromb Haemost. (2016) 115:184–92. doi: 10.1160/TH15-07-0577, PMID: 26322338

[4] Terzoudi A Vorvolakos T Heliopoulos I Livaditis M Vadikolias K Piperidou H. Sleep architecture in stroke and relation to outcome. Eur Neurol. (2009) 61:16–22. doi: 10.1159/000165344, PMID: 18948695

[5] Zhong X Gou F Jiao H Zhao D Teng J. Association between night sleep latency and hypertension: a cross-sectional study. Medicine (Baltimore). (2022) 101:e31250. doi: 10.1097/MD.0000000000031250, PMID: 36281125 PMC9592274

[6] Miano S Fanfulla F Nobili L Heinzer R Haba-Rubio J Berger M. Sas care 1: sleep architecture changes in a cohort of patients with ischemic stroke/Tia. Sleep Med. (2022) 98:106–13. doi: 10.1016/j.sleep.2022.06.002, PMID: 35816789

[7] Kim WH Yoo YH Lim JY Kang SG Jung HY Bae JN. Objective and subjective sleep problems and quality of life of rehabilitation in patients with mild to moderate stroke. Top Stroke Rehabil. (2020) 27:199–207. doi: 10.1080/10749357.2019.1673591, PMID: 31618116

[8] Habte-Gabr E Wallace RB Colsher PL Hulbert JR White LR Smith IM. Sleep patterns in rural elders: demographic, health, and Psychobehavioral correlates. J Clin Epidemiol. (1991) 44:5–13. doi: 10.1016/0895-4356(91)90195-f, PMID: 1986057

[9] Wang F Wang J Han Y Shi X Xu X Hou C. Triglyceride-glucose index and stroke recurrence in elderly patients with ischemic stroke. Front Endocrinol (Lausanne). (2022) 13:1005614. doi: 10.3389/fendo.2022.1005614, PMID: 36105408 PMC9467280

[10] Chen RL Balami JS Esiri MM Chen LK Buchan AM. Ischemic stroke in the elderly: an overview of evidence. Nat Rev Neurol. (2010) 6:256–65. doi: 10.1038/nrneurol.2010.36, PMID: 20368741

[11] Aigner A Grittner U Rolfs A Norrving B Siegerink B Busch MA. Contribution of established stroke risk factors to the burden of stroke in young adults. Stroke. (2017) 48:1744–51. doi: 10.1161/STROKEAHA.117.016599, PMID: 28619986

[12] Maaijwee NA Rutten-Jacobs LC Schaapsmeerders P van Dijk EJ de Leeuw FE. Ischaemic stroke in young adults: risk factors and long-term consequences. Nat Rev Neurol. (2014) 10:315–25. doi: 10.1038/nrneurol.2014.72, PMID: 24776923

[13] Petrov KK Hayley A Catchlove S Savage K Stough C. Is poor self-rated sleep quality associated with elevated systemic inflammation in healthy older adults? Mech Ageing Dev. (2020) 192:111388. doi: 10.1016/j.mad.2020.111388, PMID: 33080282

[14] Schloss MJ Swirski FK Nahrendorf M. Modifiable cardiovascular risk, hematopoiesis, and innate immunity. Circ Res. (2020) 126:1242–59. doi: 10.1161/CIRCRESAHA.120.315936, PMID: 32324501 PMC7185037

[15] Hawes NJ Wiggins AT Reed DB Hardin-Fanning F. Poor sleep quality is associated with obesity and depression in farmers. Public Health Nurs. (2019) 36:270–5. doi: 10.1111/phn.12587, PMID: 30761585

[16] Newton TL Fernandez-Botran R. Promoting health by improving subjective sleep quality? Reduction in depressive symptoms and inflammation as potential mechanisms and implications for trauma-exposed persons. Front Psych. (2016) 7:76. doi: 10.3389/fpsyt.2016.00076, PMID: 27199783 PMC4846648

[17] Ohayon MM Carskadon MA Guilleminault C Vitiello MV. Meta-analysis of quantitative sleep parameters from childhood to old age in healthy individuals: developing normative sleep values across the human lifespan. Sleep. (2004) 27:1255–73. doi: 10.1093/sleep/27.7.1255, PMID: 15586779

[18] Myllyntausta S Salo P Kronholm E Pentti J Oksanen T Kivimaki M. Does removal of work stress explain improved sleep following retirement? The Finnish retirement and aging study. Sleep. (2019) 42:zsz109. doi: 10.1093/sleep/zsz109, PMID: 31062863

[19] Finger CE Moreno-Gonzalez I Gutierrez A Moruno-Manchon JF McCullough LD. Age-related immune alterations and cerebrovascular inflammation. Mol Psychiatry. (2022) 27:803–18. doi: 10.1038/s41380-021-01361-1, PMID: 34711943 PMC9046462

[20] Lin YT Chen HD Ai QD Yang YT Zhang Z Chu SF. Characteristics and pathogenesis of chemokines in the post-stroke stage. Int Immunopharmacol. (2023) 116:109781. doi: 10.1016/j.intimp.2023.109781, PMID: 36720195

[21] Zaremba J Ilkowski J Losy J. Serial measurements of levels of the chemokines Ccl2, Ccl3 and Ccl5 in serum of patients with acute Ischaemic stroke. Folia Neuropathol. (2006) 44:282–9. PMID: 17183455

[22] Florholmen J Kristiansen MG Steigen SE Sorbye SW Paulssen EJ Kvamme JM. A rapid chemokine response of macrophage inflammatory protein (Mip)-1alpha, Mip-1beta and the regulated on activation, Normal T expressed and secreted chemokine is associated with a sustained Virological response in the treatment of chronic hepatitis C. Clin Microbiol Infect. (2011) 17:204–9. doi: 10.1111/j.1469-0691.2010.03206.x, PMID: 20219081

[23] Liu X Chen B Huang Z Duan R Li H Xie L. Effects of poor sleep on the immune cell landscape as assessed by single-cell analysis. Commun Biol. (2021) 4:1325. doi: 10.1038/s42003-021-02859-8, PMID: 34824394 PMC8617259

[24] Sprecher KE Koscik RL Carlsson CM Zetterberg H Blennow K Okonkwo OC. Poor sleep is associated with Csf biomarkers of amyloid pathology in cognitively Normal adults. Neurology. (2017) 89:445–53. doi: 10.1212/WNL.0000000000004171, PMID: 28679595 PMC5539733

[25] Huang WY Huang CC Chang CC Kor CT Chen TY Wu HM. Associations of self-reported sleep quality with circulating interferon gamma-inducible protein 10, interleukin 6, and high-sensitivity C-reactive protein in healthy menopausal women. PLoS One. (2017) 12:e0169216. doi: 10.1371/journal.pone.0169216, PMID: 28060925 PMC5218483

[26] Milrad SF Hall DL Jutagir DR Lattie EG Ironson GH Wohlgemuth W. Poor sleep quality is associated with greater circulating pro-inflammatory cytokines and severity and frequency of chronic fatigue syndrome/Myalgic encephalomyelitis (Cfs/me) symptoms in women. J Neuroimmunol. (2017) 303:43–50. doi: 10.1016/j.jneuroim.2016.12.008, PMID: 28038892 PMC5258835

[27] Prather AA Epel ES Cohen BE Neylan TC Whooley MA. Gender differences in the prospective associations of self-reported sleep quality with biomarkers of systemic inflammation and coagulation: findings from the heart and soul study. J Psychiatr Res. (2013) 47:1228–35. doi: 10.1016/j.jpsychires.2013.05.004, PMID: 23746737 PMC3864775

[28] Porcher L Bruckmeier S Burbano SD Finnell JE Gorny N Klett J. Aging triggers an upregulation of a multitude of cytokines in the male and especially the female rodent Hippocampus but more discrete changes in other brain regions. J Neuroinflammation. (2021) 18:219. doi: 10.1186/s12974-021-02252-6, PMID: 34551810 PMC8459490

[29] Wang Q Tang XN Yenari MA. The inflammatory response in stroke. J Neuroimmunol. (2007) 184:53–68. Epub 2006/12/26. doi: 10.1016/j.jneuroim.2006.11.014, PMID: 17188755 PMC1868538

[30] Huang Y Chen S Luo Y Han Z. Crosstalk between inflammation and the Bbb in stroke. Curr Neuropharmacol. (2020) 18:1227–36. doi: 10.2174/1570159X18666200620230321, PMID: 32562523 PMC7770647

[31] Przykaza L. Understanding the connection between common stroke comorbidities, their associated inflammation, and the course of the cerebral ischemia/reperfusion Cascade. Front Immunol. (2021) 12:782569. doi: 10.3389/fimmu.2021.782569 34868060 PMC8634336

[32] Jones KL Maguire JJ Davenport AP. Chemokine receptor Ccr5: from aids to atherosclerosis. Br J Pharmacol. (2011) 162:1453–69. doi: 10.1111/j.1476-5381.2010.01147.x, PMID: 21133894 PMC3057285

[33] Libby P. Inflammation in atherosclerosis. Nature. (2002) 420:868–74. doi: 10.1038/nature01323 12490960

[34] Kasner SE. Clinical interpretation and use of stroke scales. Lancet Neurol. (2006) 5:603–12. doi: 10.1016/S1474-4422(06)70495-1 16781990

[35] Kwah LK Diong J. National Institutes of Health stroke scale (Nihss). J Physiother. (2014) 60:61. doi: 10.1016/j.jphys.2013.12.012 24856948

[36] Reinholdsson M Palstam A Sunnerhagen KS. Prestroke physical activity could influence acute stroke severity (part of Papsigot). Neurology. (2018) 91:e1461–7. doi: 10.1212/WNL.0000000000006354, PMID: 30232251 PMC6202943

[37] Lindley RI Wardlaw JM Whiteley WN Cohen G Blackwell L Murray GD. Alteplase for acute ischemic stroke: outcomes by clinically important subgroups in the third international stroke trial. Stroke. (2015) 46:746–56. doi: 10.1161/STROKEAHA.114.006573 25613308

[38] Scinicariello F Buser MC Feroe AG Attanasio R. Antimony and sleep-related disorders: Nhanes 2005-2008. Environ Res. (2017) 156:247–52. doi: 10.1016/j.envres.2017.03.036, PMID: 28363141 PMC5685481

[39] Mai X Liang X. Risk factors for stroke based on the National Health and nutrition examination survey. J Nutr Health Aging. (2020) 24:791–5. doi: 10.1007/s12603-020-1430-4 32744577 PMC12876604

[40] Zhang Y Xia X Zhang T Zhang C Liu R Yang Y. Relationship between sleep disorders and the prognosis of neurological function after stroke. Front Neurol. (2022) 13:1036980. doi: 10.3389/fneur.2022.1036980, PMID: 36388217 PMC9659634

[41] Krishnamurthi RV Moran AE Feigin VL Barker-Collo S Norrving B Mensah GA. Stroke prevalence, mortality and disability-adjusted life years in adults aged 20-64 years in 1990-2013: data from the global burden of disease 2013 study. Neuroepidemiology. (2015) 45:190–202. doi: 10.1159/000441098, PMID: 26505983

[42] Kissela BM Khoury JC Alwell K Moomaw CJ Woo D Adeoye O. Age at stroke: temporal trends in stroke incidence in a large, biracial population. Neurology. (2012) 79:1781–7. doi: 10.1212/WNL.0b013e318270401d, PMID: 23054237 PMC3475622

[43] George MG Tong X Kuklina EV Labarthe DR. Trends in stroke hospitalizations and associated risk factors among children and young adults, 1995-2008. Ann Neurol. (2011) 70:713–21. doi: 10.1002/ana.22539, PMID: 21898534

[44] Taillard J Gronfier C Bioulac S Philip P Sagaspe P. Sleep in Normal aging, homeostatic and circadian regulation and vulnerability to sleep deprivation. Brain Sci. (2021) 11:1003. doi: 10.3390/brainsci11081003, PMID: 34439622 PMC8392749

[45] Mander BA Winer JR Walker MP. Sleep and Human Aging. Neuron. (2017) 94:19–36. doi: 10.1016/j.neuron.2017.02.004, PMID: 28384471 PMC5810920

[46] Hirshkowitz M Whiton K Albert SM Alessi C Bruni O DonCarlos L. National Sleep Foundation's updated sleep duration recommendations: final report. Sleep Health. (2015) 1:233–43. doi: 10.1016/j.sleh.2015.10.004, PMID: 29073398

[47] Hofmeijer J van Kaam R Vermeer SE van Putten M. Severely disturbed sleep in patients with acute ischemic stroke on stroke units: a pilot study. Front Neurol. (2019) 10:1109. doi: 10.3389/fneur.2019.01109, PMID: 31708856 PMC6824098

[48] Gao Y Jia Z Zhao L Han S. The effect of activity participation in middle-aged and older people on the trajectory of depression in later life: National Cohort Study. JMIR Public Health Surveill. (2023) 9:e44682. doi: 10.2196/44682, PMID: 36951932 PMC10131905

[49] Reddin C Murphy R Hankey GJ Judge C Xavier D Rosengren A. Association of Psychosocial Stress with risk of acute stroke. JAMA Netw Open. (2022) 5:e2244836. doi: 10.1001/jamanetworkopen.2022.44836, PMID: 36484991 PMC9856236

[50] McDermott M Brown DL Chervin RD. Sleep disorders and the risk of stroke. Expert Rev Neurother. (2018) 18:523–31. doi: 10.1080/14737175.2018.1489239, PMID: 29902391 PMC6300163

[51] Ding D Rogers K van der Ploeg H Stamatakis E Bauman AE. Traditional and emerging lifestyle risk behaviors and all-cause mortality in middle-aged and older adults: evidence from a large population-based Australian cohort. PLoS Med. (2015) 12:e1001917. doi: 10.1371/journal.pmed.1001917, PMID: 26645683 PMC4672919

[52] Mirabelli-Badenier M Braunersreuther V Viviani GL Dallegri F Quercioli A Veneselli E. Cc and cxc chemokines are pivotal mediators of cerebral injury in Ischaemic stroke. Thromb Haemost. (2011) 105:409–20. doi: 10.1160/TH10-10-0662, PMID: 21174009

[53] Hayaishi O. Molecular mechanisms of sleep-wake regulation: roles of prostaglandins D2 and E2. FASEB J. (1991) 5:2575–81. doi: 10.1096/fasebj.5.11.1907936, PMID: 1907936

[54] Gerozissis K De Saint HZ Orosco M Rouch C Nicolaidis S. Changes in hypothalamic prostaglandin E2 may predict the occurrence of sleep or wakefulness as assessed by parallel Eeg and microdialysis in the rat. Brain Res. (1995) 689:239–44. doi: 10.1016/0006-8993(95)00583-c, PMID: 7583327

[55] Yagami T Koma H Yamamoto Y. Pathophysiological roles of cyclooxygenases and prostaglandins in the central nervous system. Mol Neurobiol. (2016) 53:4754–71. doi: 10.1007/s12035-015-9355-3, PMID: 26328537

[56] Swift ME Burns AL Gray KL DiPietro LA. Age-related alterations in the inflammatory response to dermal injury. J Invest Dermatol. (2001) 117:1027–35. doi: 10.1046/j.0022-202x.2001.01539.x, PMID: 11710909

[57] Sieber MW Claus RA Witte OW Frahm C. Attenuated inflammatory response in aged mice brains following stroke. PLoS One. (2011) 6:e26288. doi: 10.1371/journal.pone.0026288, PMID: 22028848 PMC3196544

[58] Purroy F Vena A Forne C de Arce AM Davalos A Fuentes B. Age- and sex-specific risk profiles and in-hospital mortality in 13,932 Spanish stroke patients. Cerebrovasc Dis. (2019) 47:151–64. doi: 10.1159/000500205, PMID: 31055571

[59] Takayama K Garcia-Cardena G Sukhova GK Comander J Gimbrone MA Jr Libby P. Prostaglandin E2 suppresses chemokine production in human macrophages through the Ep4 receptor. J Biol Chem. (2002) 277:44147–54. doi: 10.1074/jbc.M204810200, PMID: 12215436

[60] Xu P Zhang S Kan X Shen X Mao J Fang C. Changes and roles of Il-17a, Vegf-a and Tnf-alpha in patients with cerebral infarction during the acute phase and early stage of recovery. Clin Biochem. (2022) 107:67–72. doi: 10.1016/j.clinbiochem.2022.05.001, PMID: 35550786

[61] Bonifacic D Toplak A Benjak I Tokmadzic VS Lekic A Kucic N. Monocytes and monocyte chemoattractant protein 1 (Mcp-1) as early predictors of disease outcome in patients with cerebral ischemic stroke. Wien Klin Wochenschr. (2016) 128:20–7. doi: 10.1007/s00508-015-0878-4, PMID: 26542133

[62] Shaafi S Sharifipour E Rahmanifar R Hejazi S Andalib S Nikanfar M. Interleukin-6, a reliable prognostic factor for ischemic stroke. Iran J Neurol. (2014) 13:70–6. PMID: 25295149 PMC4187333

